# The Scanpaths of Subjects with Developmental Prosopagnosia during a Face Memory Task

**DOI:** 10.3390/brainsci9080188

**Published:** 2019-08-02

**Authors:** Dong-Ho Lee, Sherryse L. Corrow, Raika Pancaroglu, Jason J. S. Barton

**Affiliations:** 1 Human Vision and Eye Movement Laboratory, Departments of Medicine (Neurology) and Ophthalmology and Visual Sciences, University of British Columbia, Vancouver, BC V5Z 3N9, Canada; 2 Psychology Department, Bethel University, St. Paul, MN 55112, USA

**Keywords:** face recognition, prosopagnosia, fixation, eye movements

## Abstract

The scanpaths of healthy subjects show biases towards the upper face, the eyes and the center of the face, which suggests that their fixations are guided by a feature hierarchy towards the regions most informative for face identification. However, subjects with developmental prosopagnosia have a lifelong impairment in face processing. Whether this is reflected in the loss of normal face-scanning strategies is not known. The goal of this study was to determine if subjects with developmental prosopagnosia showed anomalous scanning biases as they processed the identity of faces. We recorded the fixations of 10 subjects with developmental prosopagnosia as they performed a face memorization and recognition task, for comparison with 8 subjects with acquired prosopagnosia (four with anterior temporal lesions and four with occipitotemporal lesions) and 20 control subjects. The scanning of healthy subjects confirmed a bias to fixate the upper over the lower face, the eyes over the mouth, and the central over the peripheral face. Subjects with acquired prosopagnosia from occipitotemporal lesions had more dispersed fixations and a trend to fixate less informative facial regions. Subjects with developmental prosopagnosia did not differ from the controls. At a single-subject level, some developmental subjects performed abnormally, but none consistently across all metrics. Scanning distributions were not related to scores on perceptual or memory tests for faces. We conclude that despite lifelong difficulty with faces, subjects with developmental prosopagnosia still have an internal facial schema that guides their scanning behavior.

## 1. Introduction

Individuals with prosopagnosia are impaired in recognizing faces. This can cause awkward social interactions, such as not recognizing friends or family, resulting in feelings of guilt and avoidance of social situations [1,2]. Prosopagnosia can be a lifelong developmental problem (developmental prosopagnosia) [2,3] or acquired later in life (acquired prosopagnosia) from damage to either the anterior temporal or inferior occipital temporal cortex, most frequently in the right or both hemispheres [4]. 

A natural question about prosopagnosia is how subjects with this disorder process faces. In healthy subjects, while there is evidence that faces are processed holistically [5,6], it is also known that not all facial features contribute equally to face recognition. Instead, there is a hierarchy of feature saliency [7]. When identifying a frontally viewed face, processing its central regions (i.e., the eyes, nose and mouth) is more important than examining its external contours, and the eyes contribute more to face recognition than the other facial features [8,9,10]. This feature hierarchy is not fixed but depends on the task: while the eyes dominate when the subject is trying to recognize the person, the lower face contributes more when they are trying to identify certain expressions like happiness or disgust [10,11]. 

This feature hierarchy has been reflected in some gaze-tracking studies. A few fixations near the center of the face may suffice for rapid recognition, likely reflecting that this is an optimal point for maximum information gain [12,13]. However, the processing of a facial region is optimal when the high-resolution fovea is directed at it, and when subjects in western cultures use more time and fixations to inspect a face they show a similar feature hierarchy, with more scanning of the eyes and central features [14,15,16,17]. Additionally, mirroring the perceptual data, the distribution of fixations shifts with the goal of the task, with the upper face scanned more when the subject is identifying the face and the lower face more when judging its expression [18]. 

In addition to deficient holistic processing in acquired and developmental prosopagnosia [19,20,21,22], perceptual studies have shown a selective vulnerability of certain facial regions in these conditions. On a variety of perceptual tasks, subjects with acquired prosopagnosia have been shown to have more trouble processing the eyes than the mouth [4,23,24,25,26,27] and, likewise, in developmental prosopagnosia [28,29]. Thus, the facial areas that are the most useful for face recognition in healthy subjects may show the most severely impaired processing in prosopagnosia.

How might this inverted perceptual hierarchy be reflected in the scanning behavior while prosopagnosic subjects try to identify faces? There are several possibilities. First, subjects might scan the eyes less if eyes are less informative for them. Hence, the gaze behavior would parallel perceptual efficiency. This would be similar to the reduced scanning of relevant aspects of a scene in general visual agnosia [30]. Second, subjects might scan the eyes more to compensate for less efficient perceptual processing of this area in an effort to increase information gain. Such compensatory gaze behavior is seen in the increased contralateral scanning of hemianopic patients, for example, References [31,32]. Here gaze distribution would inversely correlate with perceptual dysfunction. Third, if the internal face schema, the feature saliency map that directs gaze, is intact despite the perceptual dysfunction that degrades face identification, then the gaze behavior of prosopagnosic subjects may be relatively normal. In this last scenario, the scanning behavior would be independent of the perceptual disorder. 

So far, the data about scanning in prosopagnosia is mixed. Among those with the acquired variant, subject PS tended to gaze at the mouth more than the eyes [33], while another subject showed a normal feature hierarchy in scanning [34]. In a recent study, we examined how 11 subjects with acquired prosopagnosia both perceived and scanned the different parts of a face while they were memorizing and then recalling faces [27]. The perceptual results depended on what type of lesion the subject had. Those with occipitotemporal lesions had more trouble discriminating the shape and position of the eyes than they did with the mouth, while those with anterior temporal lesions did not show this greater vulnerability of the ocular region. In terms of scanning behavior though, both groups tended to look only slightly less at the eyes, and at a single-subject level, this was only apparent in two subjects, one with each type of lesion. Rather, most acquired prosopagnosic subjects showed a normal bias towards the upper face and the eyes.

The fact that subjects with acquired prosopagnosia tend to scan faces in a similar manner to the controls supports the third scenario, that despite their recognition difficulties they retain an internal schema of the face that directs their gaze. This may not be surprising. After all, they know when they are looking at a face, and they have many years of experience scanning faces prior to their lesions. Hence, they may still possess a saliency map indicating the location of the most useful facial information, which generates normal scanpath strategies for extracting facial data even if their processing of that data is faulty. 

On the other hand, subjects with developmental prosopagnosia have never had normal experience with faces. Hence one might ask whether they ever developed a map of the most salient features of the face for identification. If not, their scanpaths may not show the biases seen in healthy subjects. For example, one subject with early-onset acquired prosopagnosia, a patient with epilepsy and occipital malformations, showed a shift of gaze away from the eyes to the more peripheral parts of the face [34]. On the other hand, another subject who had meningoencephalitis at age 3 had a normal bias of gaze towards the eyes and central features [35]. Although there are several potential explanations for the difference in results between these two reports, one possibility is that the internal face schema is not innate but acquired in early life.

So far, there are limited data on this point in developmental prosopagnosia. One study of four subjects and four controls reported more dispersed fixations to the facial periphery [36], though another case report did not [37]. Another study of a father and son showed fewer fixations on the eyes compared to two control subjects [38]. One group study of 10 subjects with developmental prosopagnosia as they viewed social scenes found a fixation bias for the mouth over the eyes at a group level, but with considerable heterogeneity, and did not find a difference in fixation distribution between the center and the periphery of the face [39]. More similar to our method was a study using a face-matching task, which found that a group of 12 developmental prosopagnosic subjects fixated on the mouth more than did controls [21]. Overall, some of these observations might support the lack of a normal feature hierarchy. Gaze behavior is notoriously idiosyncratic [16] and can even show cultural differences [40]. Hence confirmation of these results in both subjects and controls is needed. 

In this report, we add to our prior work on acquired prosopagnosia [27] by examining the scanning behavior of a cohort of 10 subjects with developmental prosopagnosia tested on the same memorization and retrieval task, with comparison to 20 control subjects and also with reference to our acquired prosopagnosic cohort. Our main goal was to determine if the developmental prosopagnosic subjects showed a more anomalous scanning of faces, namely, a reduction in the normal biases to look more at the upper face, the eyes, or the central regions of the face. 

## 2. Materials and Methods

### 2.1. Subjects

Prosopagnosic patients were recruited through www.faceblind.org (Table 1). The cohort included 8 subjects with acquired prosopagnosia, 4 of whom had occipitotemporal lesions (APOT, 1 female, age range 23–62 years, mean 51 years) and 4 of whom had anterior temporal lesions (APAT, 2 female, age range 25–47 years, mean 35.75 years), and 10 with developmental prosopagnosia (7 female, age range 20–52 years, mean 37.7 years). The data of our subjects with acquired prosopagnosia were previously reported [27], and are included here for comparison with the developmental cohort. Each prosopagnosic patient underwent a neuro-ophthalmological examination including Goldmann perimetry. All had a corrected visual acuity of at least 20/30 and complained of impaired face recognition in daily life. Acquired prosopagnosia was confirmed by impaired performance on at least one of two tests of face recognition, the Cambridge Face Memory Test [41] or the face component of the Warrington Recognition Memory Test [42], with normal scores on the word component of the latter. Criteria for developmental prosopagnosia included impairment on at least two of 3 tests, namely the Cambridge Face Memory Test, the face component of the Warrington Recognition Memory Test, or a famous face identification test [43]. Impairment was defined as a score of at least two standard deviations below the control means. All subjects also completed the Cambridge Face Perception Test [44] to evaluate perceptual deficits. Subjects with acquired prosopagnosia had structural and functional MRI imaging to categorize them as APOT or APAT [27]. APOTs had lesions posterior to the anterior tip of the right middle fusiform sulcus, whereas APAT was defined by anterior temporal lesions that did not extend posterior to this landmark [45]. Prior work has indicated that APOT is associated with more apperceptive deficits in face recognition than APAT, which primarily causes an amnestic variant of prosopagnosia [4]. In particular, deficits in eye perception are more characteristic of the APOT than the APAT group. As this could be theoretically reflected in differences in scanning [27], we analyzed these two subgroups of acquired prosopagnosia separately. 

All developmental prosopagnosia subjects also had neuropsychologic tests to exclude general problems with memory, intelligence, perception and attention (Table 2). This included the full-scale Wechsler Abbreviated Scales of Intelligence [46], the word lists, digit span and spatial span subtests of the Wechsler Memory Scale-III [47], and the Visual Object and Space Perception Battery [48]. DP044 did not complete the WASI and spatial span component of the Wechsler Memory Scale because she had previous experience with these tests. Because autism can be associated with impaired face recognition [49,50], we required subjects with developmental prosopagnosia to have an Autism Spectrum Quotient of 32 or less [51]. Last, to exclude any brain lesions which may mimic developmental prosopagnosia [52], seven subjects with developmental prosopagnosia had FLAIR and T1-weighted brain scans on a 3T MRI; all of which were normal. Three subjects had contraindications to MRI (DP033, DP039, DP202).

We also recruited 20 control subjects (10 female, age range 18–66 years, mean 34.4 years) of the same local community and Caucasian background as the developmental prosopagnosic cohort, who lacked any history of neurological disease or cognitive impairments, and had a corrected visual acuity of 20/30 or better.

### 2.2. Apparatus

The experiments were conducted in a dimly lit room with the subject’s face 34 cm away from a high refresh rate computer screen (140 Hz) with a 1024 × 768 pixel resolution. An Eyelink 1000 (SR Research Ltd., Mississauga, Canada) was used to track the eye movements of subjects while their head was stabilized by a chin rest. SR Research Experiment Builder 1.10.165 was used to program the experiment.

### 2.3. Protocol

Research protocols were approved by the research ethics boards at the University of British Columbia and Vancouver General Hospital. Written consent was taken from all subjects in accordance with the Declaration of Helsinki. 

**Figure 1:**
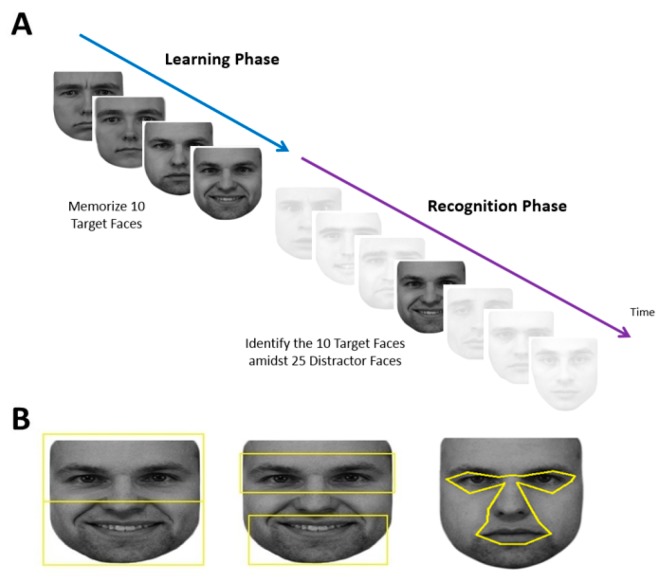
An illustration of the learning and recognition phases (**A**), the regions of interest encompassing upper versus lower regions, eye versus mouth regions, and the central region which encloses the eyes, nose and mouth (**B**).

Each subject performed a learning phase followed by a recognition phase (Figure 1A). 

Each session began with a 9-point grid fixation task to calibrate the gaze-tracker. This was followed by the learning phase, during which the subject memorized the faces of five different people, each shown twice, one at a time. The two facial images of a single person were shown consecutively with different expressions. Each face was preceded by a blank screen with a fixation cross spanning 1.43° and located 7.1° above where the next face would appear. Although having a fixation cross repeatedly above the face may bias fixations towards the lower face [53], this would affect all our comparison groups equally. Using a gaze-contingent paradigm, subjects needed to fixate within 2° of the fixation cross for at least 100 msec for the next trial to start. This addressed concerns that fixation distributions may be distorted if a trial starts with the subject’s gaze at the center of the face [12]. At the beginning of each trial, there was a blank screen that lasted 1050 msec, then the face appeared at the screen center. Subjects could study each face for as long as they wanted, at which point they pressed the spacebar to make the face disappear and to return to the fixation cross to trigger the next trial. 

At the end of the learning phase, there was a pause of up to 60 sec, which could be ended earlier if the subject pressed the space bar. This was followed by the recognition phase, which showed 35 faces—the 10 original target images and 25 new distractor images of different people—one at a time in a similar fashion. Each recognition trial began with the same 1.43° fixation cross which again needed to be fixated on for at least 100 msec. After 1050 msec of the blank screen, one of the 35 faces appeared at screen center and remained until the subject pressed either the left arrow key to indicate that they thought this was one of the five learned faces or the right arrow key to indicate that they thought it was a new distractor face. Subjects were not told how many faces would be shown in the recognition phase.

### 2.4. Stimuli

Faces used in the learning phase were taken from the KDEF Face Database [54]. Five males were randomly selected to be targets. Two faces were used for each identity, one with a neutral expression and one with either a happy or sad expression. This created 10 target faces. Twenty-five additional male facial identities were randomly selected to be distractor faces with varying emotional expressions (6 neutral, 4 happy, 5 sad, 4 surprised, 5 angry, 1 afraid). Therefore, in total there were 30 different identities shown with a total of 35 different facial images. Adobe Photoshop CS2 (www.adobe.com) was used to convert facial images to grayscale and match their luminance. The top and sides of each face were cropped to limit external features, while the natural contour of the face was used otherwise. All stimuli were presented on a white background, with the tip of the nose centered on the screen. The size of each face was set to be roughly 23° in width and 27° in height as viewed by the subject. The large size of the face image was chosen because this would increase the accuracy in classifying the location of each fixation, which was of the utmost importance in our analysis.

### 2.5. Analysis

All statistics were calculated using SPSS (Version 21, www.ibm.com) and all means are reported with standard deviation values.

We first analyzed the behavioral data for recognition accuracy, using signal detection theory to compare the sensitivity (d’) and criterion bias (c) of each group. An analysis of variance (ANOVA) was used to compare d’ and c among the 4 groups (control, developmental prosopagnosia, APAT, APOT) and post-hoc pair-wise comparisons were made with Bonferonni’s post-hoc test. 

Visual fixation data were extracted and imported into a spreadsheet. Only eye movement data when a face was present on the screen were used. Regions-of-Interests (ROIs) were created in SR Research Eyelink Data Viewer (Version 1.10.1, www.sr-research.com) to classify fixation locations. We created 6 facial ROIs in 3 paired contrasts: eyes vs. mouth, upper vs. lower face, and central vs. peripheral face. This was done for each face individually. We used the same ROIs of our previous study for the eyes vs. mouth, and upper vs. lower face [27]. The total area of the ROIs for the eyes and mouth were each 216° squared, and for the upper and lower face, 325° squared. For the central and peripheral ROIs, due to the variation in size of the central facial features versus the peripheral features in different images, the areas were comparable but not equivalent. The central ROI encompassed the eyes (but not eyebrows), nose, and mouth (Figure 1B), while the peripheral ROI was simply the remainder of the face. These were created with uniform landmarks to define each feature of the central region and extending the central boundary outwards by 0.15°, which is the margin of the position error of the Eyelink (Version 1000, www.sr-research.com). 

The scanning duration was the sum of the duration of all fixations spent in an ROI. This was calculated for each trial and averaged across trials for each subject. Because the central and peripheral ROIs differed in size, these values were divided by the ROI area to give msec per pixel. For statistical analysis, we used log (the scanning duration) because durations were skewed to larger values, particularly in the learning phase. We divided the retrieval component into two phases, one for the target and one for the distractor faces. Hence results were compared across three phases: the learning phase, a target recognition phase, and a distractor recognition phase. 

We first examined the data for the control subjects to determine if our method replicated the bias to fixate the upper face, the eyes, and the central face. For each of these, we used repeated-measures ANOVAs with phase (learning, target, distractor) and face-part (upper vs. lower, or eyes vs. mouth, or central vs. peripheral) as within-subject factors. Tests of simple main effects were delineated with the Bonferroni’s post-hoc test.

Next, we addressed a preliminary question, whether the total scan-time for the entire face differed between the groups. If developmental prosopagnosic subjects scan faces more, this could indicate a compensatory strategy—spending more time gathering information because processing that information is difficult. We used two-way ANOVAs with the group (control, developmental prosopagnosia, APAT, APOT) and phase as factors. Tests of simple main effects were analyzed and Bonferroni adjusted for post-hoc comparisons. 

For this section, we also included an analysis of the number of fixations used. However, as fixation number and scanning duration are not completely independent variables, and the analysis of one generally replicated that of the other, as in other reports [14], we limit our report on the remaining analyses to scanning duration alone. 

We then addressed our main question, whether there was a difference between our groups in the bias of fixations towards a particular part of the face. We evaluated this with three comparisons, between the upper and lower half, between the eyes and the mouth, and between the central and peripheral regions. We created an upper/lower face index by dividing the difference between the fixation durations on the upper and the lower face by the sum of the two: positive numbers indicated greater fixation to the upper half. A similar index was created for the eye/mouth contrast. For the central/peripheral contrast, we used a simple subtraction. Each of these three indices was subjected to a two-way ANOVA with the group and phase as factors. 

Additionally, to complement our central/peripheral analysis of scanning duration, and to follow a prior report of more dispersed scanning in developmental prosopagnosia [36], we derived a dispersion index to characterize how scattered a subject’s fixations were over the face. We measured the distance between each fixation and the center of the face and calculated the standard deviation of these distances for each trial. A large average standard deviation would indicate that subjects are scattering fixations broadly over a face, rather than concentrating them in a particular zone. A low average standard deviation would indicate that subjects are fixating a narrow area repeatedly. As with our other indices, the dispersion index was averaged across all trials separately for each phase for each subject. The effect of group and phase on the dispersion index was examined using a similar two-way ANOVA. 

As there may be heterogeneity within developmental prosopagnosia [28,55,56], and to ensure that group averaging did not obscure any individually anomalous patterns, we also performed a single-subject analysis for all the above indices, comparing each subject’s data to 95% prediction intervals derived from control data.

Finally, we addressed two supplementary questions. To look at the issue of possible heterogeneity further, we divided our developmental cohort into two subgroups of 5 subjects each, those with normal and those with abnormal scores on the Cambridge Face Perception Test [44]). These test indexes problems with face perception rather than face recognition, and one might hypothesize that an impaired saliency map might be more likely in those with perceptual deficits. The effects of subgroup (impaired CFPT vs. unimpaired CFPT) and phase were examined with a two-way ANOVA for each of the dependent measures listed above.

The second supplementary question was raised by an observation using the Bubbles technique that greater use of information from the eye region predicted better face recognition in healthy subjects [57]. We asked whether this would be true of scanning behavior in prosopagnosic subjects. We examined the correlation between the Cambridge Face Memory Test (CFMT) score and the eye/mouth index in the 18 prosopagnosic subjects combined across acquired and developmental groups.

## 3. Results

### 3.1. Behavioral Performance

**Figure 2:**
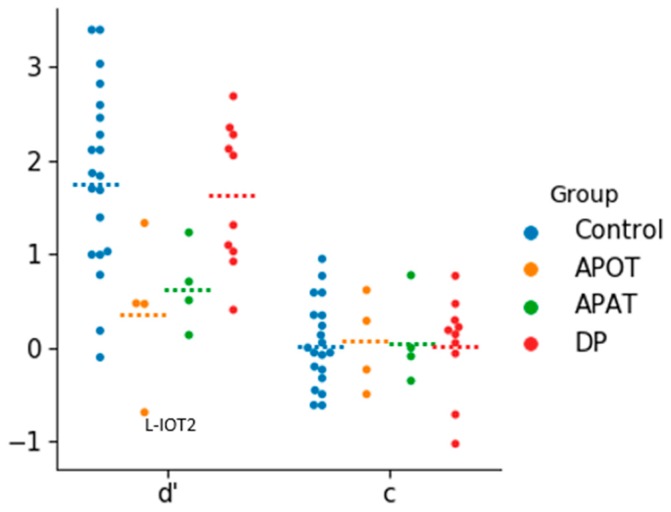
The categorical scatter plot of d’ and c values. Dotted lines represent the mean for each group. APOT = acquired prosopagnosia with occipitotemporal lesions; APAT = acquired prosopagnosia with anterior temporal lesions; DP = developmental prosopagnosia.

There were no outliers in the analysis of d’, but for c there were two outliers in the developmental prosopagnosia group. The removal of these two outliers did not impact the outcome of the analysis so they were included (Figure 2). The one-way ANOVA revealed a group effect on d’ (*F*(3,34) = 4.33, *p* = 0.011, η^2^ = 0.28). The d’ values were highest for controls (mean = 1.83 ± 0.99), followed by the developmental prosopagnosics (mean = 1.62 ± 0.76), APAT (mean = 0.64 ± 0.46) and APOT (mean = 0.40 ± 0.83) groups: post-hoc comparisons revealed a significant difference in d’ only between the APOT and control group (*p* < 0.05). For c, a one-way ANOVA revealed no significant differences between the four groups (*F*(3,34) = 0.010, *p =* 0.99, η^2^ = 0.001). 

These results suggest that our short-term recognition task is easier than standard diagnostic tests and that under these laboratory conditions the prosopagnosic subjects do not show a bias towards unfamiliarity.

### 3.2. Ocular Motor Results in Control Subjects

**Figure 3:**
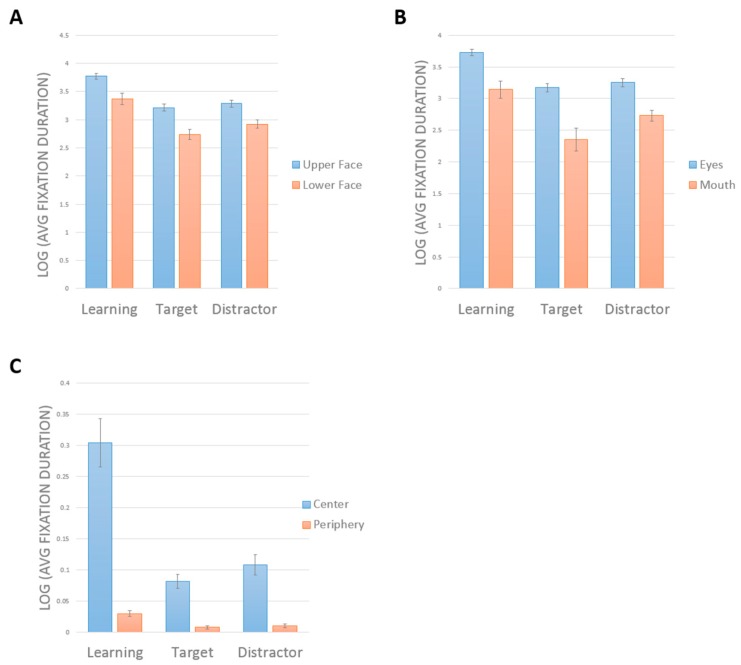
The control’s average scanning duration (log-transformed) for the learning, target, and distractor trials comparing upper versus lower face (**A**), eyes versus mouth (**B**), and central versus periphery (**C**). All error bars show the standard error of the mean.

A two-way repeated-measures ANOVA showed a main effect of face half (*F*(1,19) = 36.16, *p* < 0.001, η^2^ = 0.66). Subjects looked longer at the upper half (mean = 3.43 ± 0.05) than the lower half (mean = 3.01 ± 0.07) (Figure 3a). There was a main effect of phase (F(2,38) = 50.87, *p* < 0.001, η^2^ = 0.73). Comparisons showing a greater duration of looking during learning (mean = 3.57 ± 0.07) than in either target (mean = 2.98 ± 0.06, *p* < 0.001) or distractor phases (mean = 3.12 ± 0.06, *p* < 0.001). Subjects also looked longer in distractor than target phases (*p* = 0.034). There was no interaction between phase and face half (*F*(2,38) = 1.43, *p* = 0.25, η^2^ = 0.07).

Similar results were obtained when we compared the eye and mouth regions (Figure 3B). There was a significant interaction between phase and face part (*F*(2,38) = 4.7, *p* = 0.015, η^2^ = 0.198). Tests of main effects indicated longer scanning of the eyes than the mouth in all phases (all *p* > 0.001). There was a significant effect of phase on the scanning of the eyes (*F*(2,38) = 71.16, *p* < 0.001), with longer scanning of eyes during learning than in either the target (*p* < 0.001) or distractor phases (*p* < 0.001), but no difference between the target and distractor phases (*p* = 0.203). There was a significant effect of phase on the scanning of the mouth (*F*(2,38) = 22.874, *p* < 0.001), with longer scanning of the mouth during learning than in either the target (*p* < 0.001) or distractor phases (*p* = 0.003). However, here there was longer scanning of the mouth in the distractor than in the target phase (*p* = 0.018).

The contrast between central and peripheral ROIs showed an interaction between phase and ROI, *F*(2,38) = 33.8, *p* < 0.001, η^2^ = 0.64. Tests of simple main effects indicated longer scanning of the center than the periphery in all phases (*p* < 0.001 for all). There was a significant effect of phase on the scanning of the center, *F*(2,18) = 22.69, *p* < 0.001, with longer scanning during learning than in either the target (*p* < 0.001) or distractor phases (*p* < 0.001), but no difference between the target and distractor phases. There was a significant effect of phase on the scanning of the periphery, *F*(2,18) = 12.34, *p* < 0.001, with longer scanning of the periphery during learning than in either the target (*p* < 0.001) or distractor phases (*p* = 0.004). There was also a more scanning of the periphery in the distractor than the target phase (*p* = 0.007).

In sum, control subjects spent more time scanning in general during learning, but all three phases showed the same pattern of scanning the upper face, the eyes, and the center more. The only difference between target and distractor phases was that subjects tended to look more at the less salient regions (i.e., mouth, periphery) in the distractor trials. 

### 3.3. Ocular Motor Results: Group Comparisons of Scanning Duration and the Number of Fixations for the Entire Face

**Figure 4:**
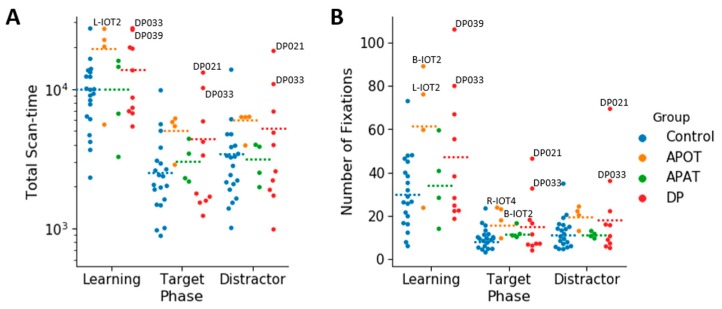
A categorical scatter plot of log(total scan-time) per face (**A**), and the number of fixations per face (**B**) in the Learning, Target and Distractor phases. The dotted lines represent the mean for each condition. Labeled subjects are abnormal by the single-subject analysis. APOT = acquired prosopagnosia with occipitotemporal lesions; APAT = acquired prosopagnosia with anterior temporal lesions; DP = developmental prosopagnosia.

For the log(total scan-time), the ANOVA showed no interaction between group and phase (*F*(6,68) = 0.168, *p* = 0.984, η^2^ = 0.015). There was no main effect of group, *F*(3,34) = 2.09, *p* = 0.119, η^2^ = 0.156). There was a main effect of phase (*F*(2,68) = 61.74, *p* < 0.001, η^2^ = 0.645). Comparisons showed longer scanning during learning (4.04 ± 0.05) than in either the target (3.50 ± 0.06), *p* < 0.001) or distractor phases (3.56 ± 0.06), *p* < 0.001) (Figure 4A). Scan time did not differ between the distractor and target phases (*p* = 0.158). 

For the total number of fixations per trial (Figure 4B), there was no interaction between group and phase (*F*(6,68) = 1.39, *p* = 0.23, η^2^ = 0.11). There was a significant effect of group (*F*(3,34) = 3.34, *p* = 0.03, η^2^ = 0.23) with comparisons showing a trend (*p* = 0.07) to more fixations by APOTs (33.55 ± 5.54) than controls (17.48 ± 2.48). There was an effect of phase (*F*(2,68) = 41.94, *p* < 0.001, η^2^ = 0.55), with more fixations during learning (43.67 ± 4.46) than in either the target (14.04 ± 1.66) or distractor phases (15.74 ± 2.34), *p* < 0.001 for both, but no difference between the target and distractor phases. 

In sum, there were no differences between the groups in how long they fixated or how many fixations they made, with the exception of a trend to APOT subjects making more fixations. 

### 3.4. Ocular Motor Results: Group Comparisons of Region of Interest Analysis

**Figure 5:**
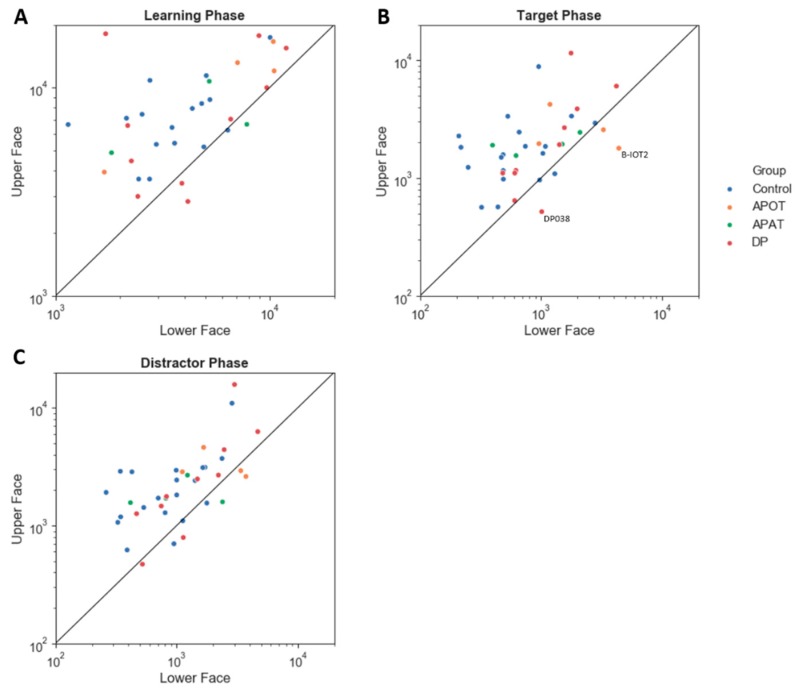
The scatter plot of the log (scanning duration) on the Upper versus Lower face, for the Learning (**A**), Target (**B**) and Distractor phases (**C**). The straight line shows an equal duration of fixation on the Upper versus Lower face. Points above the line indicate longer fixation duration on the upper face. Labeled subjects are abnormal by the single-subject analysis. APOT = acquired prosopagnosia with occipitotemporal lesions; APAT = acquired prosopagnosia with anterior temporal lesions; DP = developmental prosopagnosia.

The upper/lower index showed no interaction between group and phase (*F*(6,68) = 0.82, *p* = 0.56, η^2^ = 0.07). There was no main effect of group (*F*(3,34) = 1.51, *p* = 0.23, η^2^ = 0.12) or phase (*F*(2,68) = 0.21, *p* = 0.81, η^2^ = 0.01). All groups showed a preference for the upper face (Figure 5). All group pair-wise comparisons can be seen in Supplementary Table S1.

**Figure 6:**
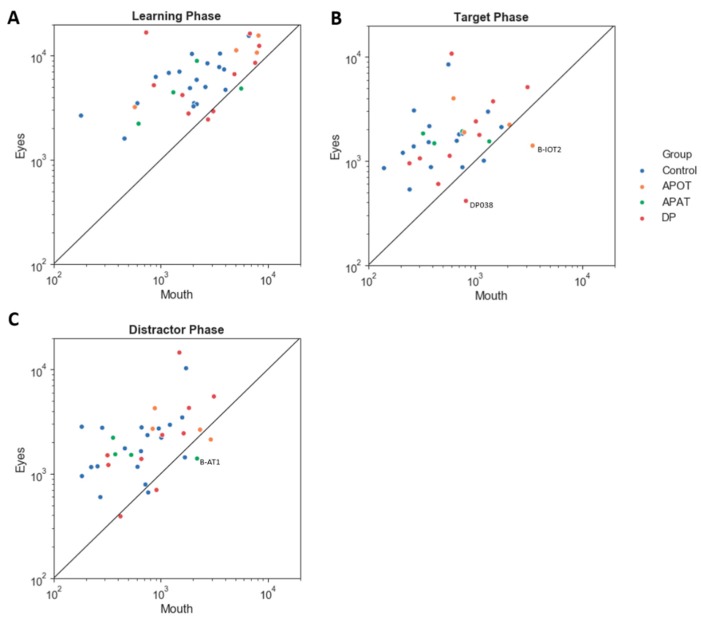
The scatter plot of log (scanning duration) on the eye versus mouth region, in the Learning (**A**), Target (**B**) and Distractor phases (**C**). The straight line shows an equal duration of fixation on the eye versus the mouth; points above this indicate more fixation time on the eyes. Labeled subjects are abnormal by the single-subject analysis. APOT = acquired prosopagnosia with occipitotemporal lesions; APAT = acquired prosopagnosia with anterior temporal lesions; DP = developmental prosopagnosia.

For the eye/mouth index, there was no interaction between group and phase (*F*(6,68) = 1.24, *p* = 0.30, η^2^ = 0.10) and no main effect of group (*F*(3,34) = 1.39, p = 0.26, η^2^ = 0.11) or phase (*F*(2,68) = 0.17, *p* = 0.85, η^2^ = 0.01). All groups fixated on the eyes more than the mouth (Figure 6). 

**Figure 7:**
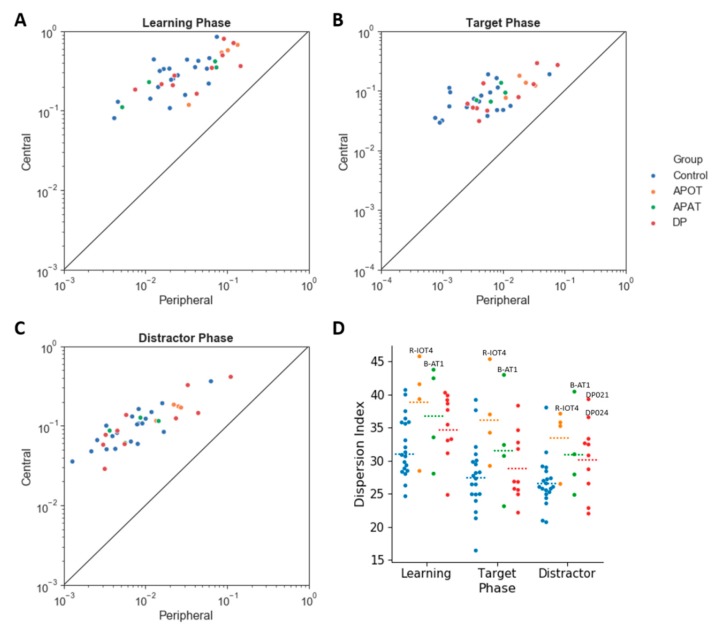
The scatter plot of log (scanning duration) on the central versus peripheral region, in the Learning (**A**), Target (**B**) and Distractor phases (**C**). The straight line shows equal duration of fixation on the center versus the periphery; points above this indicate more fixation time on the center. Categorical scatter plot of the dispersion index (**D**) in the Learning, Target and Distractor phases. Dotted lines represent the mean for each condition. Labeled subjects are abnormal by the single-subject analysis. APOT = acquired prosopagnosia with occipitotemporal lesions; APAT = acquired prosopagnosia with anterior temporal lesions; DP = developmental prosopagnosia.

For the central/peripheral index (Figure 7) there was no interaction between group and phase (*F*(6,68) = 1.36, *p* = 0.24, η^2^ = 0.11) and no main effect of phase (*F*(2,68) = 0.77, *p* = 0.47 η^2^ = 0.02). There was a main effect of group (*F*(3,34) = 3.15, *p* = 0.04, η^2^ = 0.22). Post hoc comparisons found a trend (*p* = 0.07) to lower indices in APOTs (6.65 ± 4.02) than compared to Controls (18.28 ± 1.8). Thus, all groups showed a bias towards central fixations (Figure 7A), with a trend to this being reduced in the APOT group.

For the dispersion index (Figure 7D), there was no interaction between group and phase (*F*(6,68) = 0.69, *p* = 0.66, η^2^ = 0.06) but a significant main effect of group (*F*(3,34) = 3.86, *p* = 0.02, η^2^ = 0.25) with larger dispersion indices in APOTs (36.27 ± 2.25) than in Controls (28.78 ± 1.01), *p* = 0.03. There was a main effect of phase (*F*(2,68) = 19.87, *p* < 0.001, η^2^ = 0.37) with larger dispersion indices during learning (35.79 ± 1.05) than in either the target (31.31 ± 1.14) or distractor phases (30.45 ± 0.94, *p* < 0.001 for both). There was no difference between the target and distractor phases (*p* = 0.73).

### 3.5. Single-Subject Analysis

Among those with acquired prosopagnosia, three of four subjects with occipitotemporal lesions used more fixations to scan faces on one or more phases (B-IOT2, LIOT2, RIOT4), while none of those with anterior temporal lesions did. However, only B-IOT2 showed a reduced tendency to scan the eyes and upper face, and only in the target phase, while R-IOT4 showed more dispersed fixations. Among those with anterior temporal lesions, the only abnormal results belonged to B-AT1, who showed reduced scanning of the eyes for distractors, and more dispersed fixations. In general, these results corroborate the group analysis. 

Among those with developmental prosopagnosia, three used more fixations than controls (DP033, DP021, DP039), but none of these three showed abnormal indices of regional scanning in the ROI analysis, except for more dispersed scanning by DP021. Of the rest, DP038 showed reduced scanning of the eyes but only in the target phase and, like DP021, DP024 showed more dispersed fixations. No subject showed a reduced central/peripheral index. 

### 3.6. Comparison of Developmental Prosopagnosia Subjects with and without Face Discrimination Impairments

For the log (the scanning duration) per trial, there was a slight violation of the assumption of normality for the unimpaired group, for the learning trials only (Shapiro–Wilk’s p = 0.02). As ANOVA is fairly robust to violations of normality, we proceeded with the analysis. The ANOVA showed no interaction or main effect of the subgroup on log (the scanning duration) (Table 3). Similarly, the results for the analyses of indices of biased scanning did not show any main effects or interactions of the subgroup (Table 3). 

### 3.7. Correlation between Eye/Mouth Index and CFMT Score

A Pearson’s correlation assessed the relationship between the eye/mouth index and the score on the Cambridge Face Memory Test for each of the three phases separately, combining the data for all prosopagnosic subjects. This relationship was not significant for the learning (r(18) = 0.035, *p* = 0.891), target (r(18) = 0.091, *p* = 0.718), or distractor phases (r(18) = 0.041, *p* = 0.872). 

## 4. Discussion

Overall, the main impression from our results is that, as a group, subjects with developmental prosopagnosia show fairly normal scanning of faces when they are memorizing and identifying faces, similar to what we previously reported for our subjects with acquired prosopagnosia. Among the latter, there are some mild differences in the group with occipitotemporal lesions. This group showed a trend to use more fixations, a trend to scan the periphery more than the center of the faces, and more dispersal of fixations across the face. These results must be treated with caution given the small numbers involved. In contrast, no significant differences were found between the controls and either those with developmental prosopagnosia or those who acquired prosopagnosia from anterior temporal lesions. 

There is evidence that, at least for face identification, the eyes are the most informative part of the face [8,9,10]. In healthy subjects, the Bubbles technique showed that face recognition ability correlates with greater dependence of recognition on the eyes [29] and that 20% of the variance in the face recognition ability of healthy subjects could be explained by contributions from the eye region [57]. This is reflected in scanning biases, with healthy subjects scanning the eyes more than the other facial features [14,15,16,17,18]. In our study, the bias to scan the eyes and upper face was similar in all three groups. We had previously reported the persistence of this bias in the acquired prosopagnosic subjects of this study, which is consistent with two prior cases [34,35] but not another [33]. Reduced scanning of the eyes was one aspect of the disorganized scanning of a subject with congenital prosopagnosia associated with epilepsy and occipital polymicrogyria [34], but this subject also had more profound visual dysfunction [52]. A similar reduction in eye-scanning bias was noted in two related subjects with developmental prosopagnosia being tested for recognition of personally familiar faces [38], and there was a trend to more scanning of the mouth in a group of 12 developmental prosopagnosic subjects as they performed a face-matching task [21]. On the other hand, a study of 10 developmental prosopagnosic subjects viewing social scenes found a tendency to scan the eyes more than the mouth at a group level, but at a single-subject level, only two scanned the mouth more than controls and none scanned the eyes less [39]. Given the limitation of these small samples, our result is not that different from the latter. With 10 developmental prosopagnosic subjects and 20 controls, we found no reduction of the eye-scanning bias at a group level and only one subject with a reduction in only one of the three phases analyzed. Thus, despite lifelong problems with face recognition, our evidence suggests that developmental prosopagnosic subjects show a normal bias towards looking at the eyes. 

Our APOT subjects showed more dispersed fixations and a trend to less emphasis on fixations on the center of the face. All of these subjects had damage to the right fusiform face area [27], which shows greater activation on functional imaging when subjects are viewing the central face region [58]. In contrast, the fusiform face area is spared by definition in our APAT cohort [27] and studies generally show persistent activation of the fusiform face area in developmental prosopagnosia [59]. Hence, one might be tempted to draw a link between structure (damage to the right fusiform face area) and function (reduced scanning of the central face). However, functional imaging studies also show more activation of the fusiform face area by the eyes [60], and yet we did not find a reduced eye-scanning bias in our APOT group. While we did find a weak tendency for reduced eye scanning in our prior report [27], this was also seen in the APAT group there. Hence, inferences from modest trends in scanning data regarding the functional integrity of the fusiform face area are speculative at best. 

Regardless, we also report no difference in central/peripheral biases in our developmental cohort. This is also in agreement with the finding of the study using social scenes [39]. With a related metric, a previous study of four subjects with hereditary prosopagnosia, three of whom were related, found more dispersed scanning compared to four controls [36]. We found more dispersed scanning in our APOT group but not in our developmental cohort, though the single-subject analysis found more dispersed scanning in two developmental subjects. While task differences and small sample sizes are always potential sources of discrepant results, one could also speculate that such differences are valid and suggestive of heterogeneity in developmental prosopagnosia [55,56,61]. 

Is there any other evidence from our scanning data to support heterogeneity within developmental prosopagnosia? Although our group results did not differ from controls, the single-subject analysis showed occasional subjects with abnormal results. However, there were no subjects that showed abnormalities in more than one metric, and abnormal individual results were not limited to one particular metric. Hence, these variations may just reflect chance. Could scanning differences be related to perceptual ability? This seems unlikely. Of the six subjects who showed at least one abnormal scanning result, only one (DP024) performed poorly on the Cambridge Face Perception Test [44]. Likewise, our analysis of subgroups within developmental prosopagnosia did not show any differences in any scanning bias between those with and those without impairments on the Cambridge Face Perception Test.

The lack of correlation between performance on the Cambridge Face Memory Test and the eye/mouth scanning index is of interest. A prior perceptual study of a large sample of 112 healthy subjects, including eight face super-recognizers and five with developmental prosopagnosia, found that the more informative the eye region is to a subject, the better their performance on this test of face recognition [29]. Within our impaired group though, we found no correlation of those test scores with an eye-scanning bias. One might argue that it is not known whether scores on the Cambridge Face Memory Test accurately capture the severity of the recognition deficit in prosopagnosia. Such a result may also underline important differences between analyzing perceptual processing and assessing scanning behavior, particular in pathologic states. As outlined in the introduction, it is not logically necessary that regional reductions in perceptual efficiency are paralleled by similar reductions in scanning. As our prior study demonstrated [27], perceptual discrimination and scanning distribution show independent regional effects in acquired prosopagnosia. 

In summary, our results show that, as a group, only our subjects with acquired prosopagnosia from occipitotemporal lesions showed more dispersed scanpaths and a trend to less emphasis on the central face. Our main result is that those with developmental prosopagnosia showed normal scanning biases for the upper face, the eyes and the central face, and did not show greater dispersion of fixations. Such results suggest that despite lifelong difficulties with recognizing faces, these subjects retain a normal facial schema that directs their scanpaths towards the more informative regions for face identification.

Our study is not without limitations. First, though we report results for a much larger sample of developmental prosopagnosic subjects and controls than prior studies, the numbers are still modest, and more subtle effects may emerge from a larger study. Second, we did not match the IQ of our prosopagnosia subjects with the control subjects. Although IQ is an important factor in cognitive studies, we did not find experiments suggesting a normal range of IQ had effects on fixations during face recognition. Furthermore, in previous studies in which we measured the IQ of our developmental prosopagnosia and control subjects, we found no difference between groups [1]. Third, we did not one-to-one match for gender. Although there is evidence gender can affect eye movement, our sample did not have an extraordinary gender bias (3/8 in the AP group, 7/10 in the DP group and 10/20 in the control group were female), and thus the effect of gender is likely minimized. Fourth, for our single-subject comparisons, we did not use a Bonferroni correction as it comes with a dramatic reduction of power for 20 subjects. However, with a sample of this size, we would expect at approximately one case of type I error in each of the single-subject analyses. However, for many of the comparisons, we found several impaired subjects. Still, this should be taken into account when interpreting these results. Finally, we opted to use faces of varied emotional expressions in our recognition task but the emotions in the target faces were not exactly matched with the emotions used in our distractor faces. Therefore, it is possible that subjects could have used the emotional expressions as a clue in their task. This may preference subjects to look towards the mouth [18] and may have affected our behavioral results. However, the emotional expressions were not prominent features in the images (Figure 1) nor were they mentioned in the task instructions, therefore any effect would likely be minimal and affect all three groups relatively equally. 

ur analysis focused on a simple spatial analysis of fixation distribution, yet fixations are made in a sequential pattern. Analysis of scanning temporal dynamics is more complex but may show organizational aspects that are not evident in a spatial analysis. Indeed, this has been used to show more chaotic scanning despite the preservation of normal feature biases in one patient with acquired prosopagnosia [34]. Hence, while the current report establishes that the normal feature hierarchy is still evident in scanning behavior in developmental prosopagnosia, future studies are needed to clarify whether their perceptual problems are reflected in their facial scanpaths in other metrics. 

## 5. Conclusions

Overall, our study did not find differences in the fixations between developmental prosopagnosia and control subjects, except at a single-subject level. However, our study was able to confirm the bias healthy subjects have for the upper half of the face versus the lower, the eyes over the mouth, and the central region over the periphery. In subjects with acquired prosopagnosia secondary to occipitotemporal lesions, fixations were more dispersed and there was a trend to less informative areas of the face. These scan patterns were not correlated to scores from face perception and memory tests. These results suggest that subjects with developmental prosopagnosia may have an internal face schema to guide their scanning behavior.

## Figures and Tables

**Table 1:** Demographic, inclusion and clinical information for all prosopagnosic patients. Bold values with * indicate impaired face processing. APOT = acquired prosopagnosia with occipitotemporal lesion; APAT = acquired prosopagnosia with anterior temporal lesion; DP = developmental prosopagnosia; CFMT = Cambridge Face Memory Test; WRMT = Warrington Recognition Memory Test; CFPT = Cambridge Face Perception Test; LUQ = left upper quadrant; BHH = bilateral homonymous hemianopia.

**APOTs**	**Age**	**Gender**	**Visual Field**	**Lesion**	**CFMT (/72)**	**WRMT (Face/50)**	**WRMT (Words/50)**	**CFPT**
R-IOT4	62	M	LUQ	Infarction	27*	39	50	76*
B-IOT2	60	M	BHH	Subdural hematoma	24*	21*	42	70*
L-IOT2	59	M	Full	L fusiform resection, R atrophy	21*	27*	42	74*
B-ATOT2	23	F	Full	HSV Encephalitis	24*	19*	50	80*
**APATs**	**Age**	**Gender**	**Visual Field**	**Lesion**	**CFMT (/72)**	**WRMT (Face/50)**	**WRMT (Words/50)**	**CFPT**
R-AT2	34	F	Full	HSV Encephalitis	33*	27*	47	40
R-AT3	37	M	Full	HSV Encephalitis	31*	31*	47	62*
B-AT1	25	M	Full	HSV Encephalitis	30*	27*	45	48
B-AT2	47	F	Full	Trauma, temporal resection	31*	31*	46	76*
**DPs**	**Age**	**Gender**	**Visual Field**	**Famous Faces (/60)**	**CFMT (/72)**	**WRMT (Face/50)**	**WRMT (Words/50)**	**CFPT**
DP014	42	M	Full	8*	32*	30*	48	64*
DP016	52	F	Full	37*	41*	37*	49	48
DP021	29	F	Full	25*	37*	33*	50	36
DP024	35	F	Full	14*	41*	38*	50	62*
DP033	46	F	Full	32*	29*	39	50	52
DP035	40	M	Full	9*	36*	35*	49	86*
DP038	27	F	Full	N/A	39*	36*	50	32
DP044	36	F	Full	26*	40*	34*	49	68*
DP039	50	M	Full	37*	22*	46	50	54
DP202	20	F	Full	N/A	33*	32*	50	64*

**Table 2:** The neuropsychological results for Developmental Prosopagnosia subjects. Bold scores indicate impaired performance. NA = Not administered; WASI = Wechsler Abbreviated Scales of Intelligence; VOSP = Visual Object Space Perception Battery; AQ = Autism Spectrum Quotient.

	DP014	DP016	DP021	DP024	DP033	DP035	DP038	DP039	DP044	DP202
**WASI-Full IQ**	129	120	107	137	135	116	120	117	NA	130
**Wechsler Memory Scale**										
Word Lists I	13	8	17	16	16	13	17	9	13	8
Word Lists II	13	11	12	15	15	13	13	8	15	9
Digit Span	14	14	10	14	12	16	10	11	16	10
Spatial Span	17	14	6	16	12	15	12	11	NA	9
**VOSP**										
Silhouettes	14	20	21	22	21	20	23	21	22	22
Object Decision	19	17	18	18	17	20	19	18	15	16
Progressive Silhouettes	8	10	9	6	11	11	11	8	10	8
AQ	30	16	12	22	15	15	25	13	25	22

**Table 3:** The results of the 2 (subgroup: impaired CFPT v. unimpaired CFPT) x 3 (phase: learning, distractor, target) ANOVA for each of the dependent variables (left column). * = Greenhouse-Geisser corrected.

	Subgroup x Phase Interaction	Main Effect of Subgroup
**Full Face**	*F*(2,16) = 0.088*, *p* = 0.792, η^2^ = 0.011	*F*(1,8) = 2.3, *p* = 0.168, η^2^ = 0.224
**Upper/Lower**	*F*(2,16) = 0.944*, *p* = 0.369, η^2^ = 0.106	*F*(1,8) = 0.249, *p* = 0.631, η^2^ = 0.03
**Eye/Mouth**	*F*(2,16) = 0.491*, *p* = 0.537, η^2^ = 0.058	*F*(1,8) = 0.106, *p* = 0.753, η^2^ = 0.13
**Central/Peripheral**	*F*(2,16) = 0.016*, *p* = 0.912, η^2^ = 0.002	*F*(1,8) = 0.402, *p* = 0.544, η^2^ = 0.048
**Dispersion Index**	*F*(2,16) = 0.131, *p* = 0.878, η^2^ = 0.016	*F*(1,8) = 0.546, *p* = 0.481, η^2^ = 0.064

## Supplementary Materials

The following are available online at https://www.mdpi.com/2076-3425/9/8/188/s1, Table S1. Pairwise p-value comparisons for each between-group comparison ANOVA. inclusion and clinical information for all prosopagnosic patients.
