# Supplementary material for: The Scanpaths of Subjects with Developmental Prosopagnosia during a Face Memory Task
Source: Brain Sci. 2019 Aug 2;9(8):188. doi: 10.3390/brainsci9080188 (PMC6721422; doi:10.3390/brainsci9080188)
Supplement: Supplementary file 1 [file brainsci-09-00188-s001.pdf]

Supplementary Table S1. Pairwise p-value comparisons for each between-group comparison ANOVA. inclusion and clinical information for all prosopagnosic patients. Bold values show statistically significant p-values ($p < 0.05$). Values with # adjacent represent trends ($0.05 < p < 0.10$). APOT = acquired prosopagnosia with occipitotemporal lesion; APAT = acquired prosopagnosia with anterior temporal lesion; DP = developmental prosopagnosia.

**Upper versus Lower Face Index**				
	**APOT**	**APAT**	**DP**	**Control**
APOT	X			
APAT	1.000	X		
DP	1.000	1.000	X	
Control	0.69	1.000	0.571	X
**Eye versus Mouth Index**				
	**APOT**	**APAT**	**DP**	**Control**
APOT	X			
APAT	1.000	X		
DP	1.000	1.000	X	
Control	.821	1.000	.616	X
**Central versus Periphery Index**				
	**APOT**	**APAT**	**DP**	**Control**
APOT	X			
APAT	1.000	X		
DP	1.000	1.000	X	
Control	0.074#	1.000	0.252	X
**Dispersion Index**				
	**APOT**	**APAT**	**DP**	**Control**
APOT	X			
APAT	1.000	X		
DP	.522	1.000	X	
Control	**0.027***	.415	0.708	X
**Total Scan-Time**				
	**APOT**	**APAT**	**DP**	**Control**
APOT	X			
APAT	.744	X		
DP	1.000	1.000	X	
Control	.162	1.000	.958	X
**Number of Fixations**				
	**APOT**	**APAT**	**DP**	**Control**
APOT	X			
APAT	.519	X		
DP	1.000	1.000	X	
Control	0.0735#	1.000	0.181	X

**Group Comparisons by Phase**				
			**Phases**	
**Index Type**	**Group Comparisons**	**Learning**	**Target**	**Distractor**
Upper versus Lower Face Index				
	Control vs APOT	1.000	.289	1.000
	Control vs APAT	1.000	1.000	1.000
	Control vs DP	.453	.610	1.000
	APOT vs APAT	1.000	1.000	1.000
	APOT vs DP	1.000	1.000	1.000

	APAT vs DP	1.000	1.000	1.000
Eye versus Mouth Index				
	Control vs APOT	1.000	.268	1.000
	Control vs APAT	1.000	1.000	1.000
	Control vs DP	.392	.597	1.000
	APOT vs APAT	1.000	1.000	1.000
	APOT vs DP	1.000	1.000	1.000
	APAT vs DP	1.000	1.000	1.000
Central versus Periphery Index				
	Control vs APOT	0.476	.265	0.257
	Control vs APAT	1.000	1.000	1.000
	Control vs DP	1.000	.234	1.000
	APOT vs APAT	1.000	1.000	1.000
	APOT vs DP	1.000	1.000	1.000
	APAT vs DP	1.000	1.000	1.000
Dispersion Index				
	Control vs APOT	.163	**0.043***	**0.0586***
	Control vs APAT	.625	.830	.569
	Control vs DP	.762	1.000	.239
	APOT vs APAT	1	1.000	1
	APOT vs DP	1	.189	1
	APAT vs DP	1.000	1.000	1.000
Total Scan-time				
	Control vs APOT	.444	.251	.430
	Control vs APAT	1.000	1.000	1.000
	Control vs DP	1.000	1.000	1.000
	APOT vs APAT	.861	1.000	1.000
	APOT vs DP	1.000	1.000	1.000
	APAT vs DP	1.000	1.000	1.000
Number of Fixations				
	Control vs APOT	0.082#	0.339	1.000
	Control vs APAT	1.000	1.000	1.000
	Control vs DP	.462	0.427	.619
	APOT vs APAT	0.597	1.000	1.000
	APOT vs DP	1.000	1.000	1.000
	APAT vs DP	1.000	1.000	1.000
